# Features of lumbar spine texture extracted from routine MRI correlate with bone mineral density and can potentially differentiate patients with and without fragility fractures in the spine

**DOI:** 10.1590/1414-431X2023e12454

**Published:** 2023-02-27

**Authors:** J.G. Maciel, C.E.G. Salmon, B.S. Hosseini, P.M. Azevedo-Marques, F.J.A. de Paula, M.H. Nogueira-Barbosa

**Affiliations:** 1 Departamento de Imagens Médicas, Hematologia e Oncologia Clínica, Faculdade de Medicina de Ribeirão Preto, Universidade de São Paulo, Ribeirão Preto, SP, Brasil; 2 Departamento de Física, Faculdade de Filosofia, Ciências e Letras de Ribeirão Preto, Universidade de São Paulo, Ribeirão Preto, SP, Brasil; 3 Departamento de Clínica Médica, Faculdade de Medicina de Ribeirão Preto, Universidade de São Paulo, Ribeirão Preto, SP, Brasil; 4 Department of Orthopedic Surgery, University of Missouri Health Care, Columbia, MO, USA

**Keywords:** Osteoporosis, Fracture, Texture attributes, Magnetic resonance imaging, Bone mineral density

## Abstract

The use of routine magnetic resonance imaging (MRI) to potentially assess skeletal fragility has been widely studied in osteoporosis. The aim of this study was to evaluate bone texture attributes (TA) from routine lumbar spine (LS) MRI and their correlation with vertebral fragility fractures (VFF) and bone mineral density (BMD). Sixty-four post-menopausal women were submitted to LS densitometry, total spine radiographs, and routine T2-weighted LS MRI. Twenty-two TA were extracted with the platform IBEX from L3 vertebra. The statistical difference was evaluated using ANOVA and Duncan's post-test. Correlation analyses were performed using Spearman's coefficient. Statistical significance was considered when P<0.05. The results did not show a significant difference in BMD between the women with and without fractures. Two bone TA (cluster tendency and variance) were significantly lower in the fracture group. Cluster tendency with VFF in osteopenia was 1.54±1.37 and in osteoporosis was 1.11±58. Cluster tendency without VFF in osteopenia was 2.23±1.38 and in osteoporosis was 1.88±1.14). Variance with VFF in osteopenia was 1.44±1.37 and in osteoporosis was 1.13±59. Variance without VFF in osteopenia was 2.34±1.38 and in osteoporosis was 1.89±1.14. There was a significant correlation between BMD and cluster prominence (r=0.409), cluster tendency (r=0.345), correlation (r=0.570), entropy (r=0.364), information measure corr1 (r=0.378), inverse variance (r=0.449), sum entropy (r=0.320), variance (r=0.338), sum average (r=-0.274), and sum variance (r=-0.266). Our results demonstrated the potential use of TA extracted from routine MRI as a biomarker to assess osteoporosis and identify the tendency of skeletal fragility vertebral fractures.

## Introduction

Osteoporosis is a chronic osteometabolic disease characterized by reduced bone mineral density (BMD), deterioration of bone microarchitecture, and increased susceptibility to fractures (1). Fragility fractures are an important cause of morbidity and mortality in the elderly, impairing the quality of life and survival in this population (2). The spine is commonly affected by these types of fractures; however, due to the nonspecific clinical presentation, vertebral fractures are often underdiagnosed in clinical practice, leading to an increased risk of other fragility fractures in subsequent years (3).

Dual X-ray absorptiometry (DXA or DEXA) is the gold standard technique for detecting low bone mass, assess fracture risk, and monitor treatment response (1). Bone mineral density (BMD) measured by DXA correlates with skeletal fragility and fracture risk (4). However, population-based studies have demonstrated that many of the individuals who experience a fragility fracture have BMD values above the threshold established by the World Health Organization (WHO) for the diagnosis of osteoporosis (5,6). In addition, most of the individuals at risk for fragility fractures have never been screened by DXA: up to 80% of individuals who sustain a fragility fracture report never having been evaluated or treated for osteoporosis. Moreover, DXA is still used as an initial but insufficient step in the evaluation of the efficacy of new drugs (7,8). This has encouraged the development of other diagnostic techniques that can reveal impaired bone resistance (9). Several studies have demonstrated the potential use of magnetic resonance imaging (MRI) to detect alterations in other parameters of bone quality that could indicate skeletal fragility partially explained by BMD. The detection of bone structural deterioration and compositional changes with aging has been implemented in routine MRI screening (10,11).

Bone marrow is predominantly composed of hematopoietic cells, adipocytes, and water. The increase in lipid content within bone marrow with aging is the main determinant of its signal intensity and texture heterogeneity (12-14). The analysis of the texture of a tissue corresponds to the evaluation of the distribution of gray levels within a region and reflects the uniformity and homogeneity within the evaluated region (15). The gray level co-occurrence matrix (GLCM) texture analysis proposed by Haralick et al. (16) in 1973 has been widely used in the last decades for the evaluation of texture of different organs and tissues. A few publications have demonstrated its application in bone tissue, such as a study published by Maciel et al. (17) that used this model to demonstrate a significant correlation between bone texture features from routine MRI sequences and bone mass.

Because MRI is an exam commonly used to assess low back pain (18), the use of this imaging technique to simultaneously assess BMD and skeletal fragility may benefit patients who undergo this imaging procedure for other diagnostic reasons. In this context, our objective was to evaluate bone texture features extracted from routine lumbar spine MRI sequences and assess their correlation with BMD and fragility fractures in the spine. We aimed to identify potential measurable bone texture features that could differentiate post-menopausal women with and without fragility fractures.

## Material and Methods

### Study design

This was a prospective study and included 64 post-menopausal women recruited from the Osteometabolic Diseases Outpatient Clinic of Ribeirao Preto Medical School Hospital. Individuals from the control group were recruited through posters placed on the hospital walls. Our subjects were divided into five groups: normal bone mass (n=16), which consisted of our control group, osteopenia without vertebral fragility fractures (VFF, n=12), osteopenia with VVF (n=12), osteoporosis without VFF (n=12), and osteoporosis with VFF (n=12).

### Inclusion and exclusion criteria

The inclusion criteria were post-menopausal women over 50 years of age. The exclusion criteria were: history of neoplasia, current or treated; history of osteometabolic bone diseases other than osteoporosis; current antiresorptive therapy or medications that may affect bone metabolism. We also excluded from our analysis any vertebral bodies with bone lesions or structural abnormalities that could affect bone texture.

### Image acquisition

#### Spine radiograph protocol

All volunteers underwent radiographs of the spine (T4 to S1) in the anteroposterior and lateral views. The images were analyzed by a musculoskeletal radiologist (JGM) who assessed the presence and severity of vertebral fractures using a semiquantitative visual technique as described by Genant et al. (19) and Wáng et al. (20) and classified them as discrete (grade 1: 20-25% height reduction), moderate (grade 2: 26-40% height reduction), or severe (grade 3: more than 40% height reduction).

### Bone densitometry protocol

All volunteers were submitted to DXA of the lumbar spine (L1-L4) with BMD measurement (g/cm^2^). The exams were performed using a Hologic Discovery DXA system (CI/WI, 4500W/CE, USA). The volunteers were classified into three groups according to T-score using the World Health Organization (WHO) criteria. By convention, a score above or equal to -1 is considered normal, a score between -1 and -2.5 indicates osteopenia, and a score below or equal to -2.5 indicates osteoporosis (1). A musculoskeletal radiologist (JGM) analyzed the DXA images and excluded any vertebrae with fractures or structural alterations that could compromise the results of the present analysis.

### Lumbar spine MRI acquisition protocol

All MRI examinations were performed using a 3.0-Tesla ACHIEVA MRI (Philips Medical System, USA) using a phased-array coil for the lumbar region. T2-weighted sagittal fast spin-echo (FSE) sequence of the lumbar spine was acquired with the following parameters: TR/TE: 3795/120 ms; FOV: 180×320×55 mm; slice thickness: 4.2 mm; gap: 4.4 mm; NSA: 2; scanning time: 1:45 min; and the number of slices: 12.

### Vertebral texture analysis

#### Segmentation of L3 vertebra

**Figure 1:**
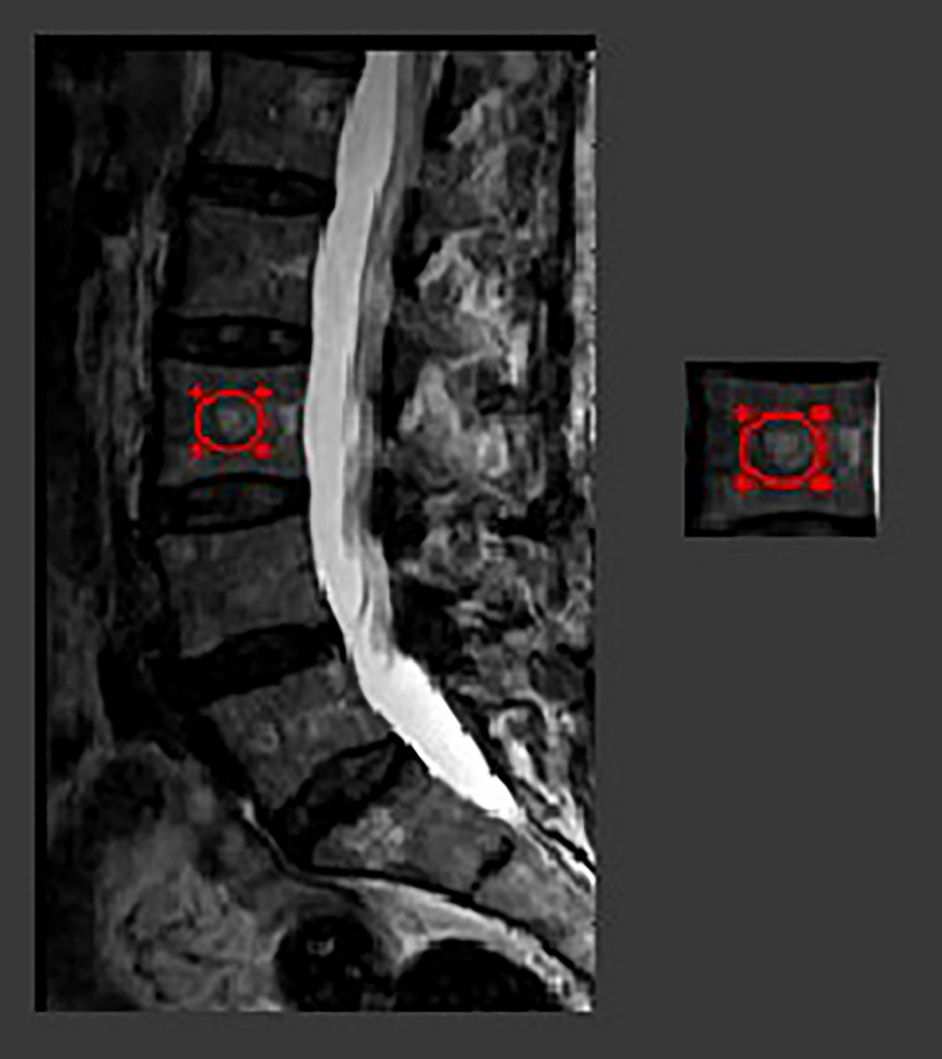
Image demonstrating the region of interest positioning in the central region of L3 vertebra on the sagittal T2-weighted fast spin echo MRI sequence.

Texture features were extracted from the L3 vertebra through the positioning of a circular region of interest (ROI) in the central portion of the vertebral body, as illustrated in Figure 1. A standardized, circular ROI (10 mm diameter) was placed in three consecutive slices, the first one in the midsagittal slice and the other two in two adjacent slices. For the analysis, the means and standard deviations of the values extracted from the 3 consecutive ROIs were calculated. In only one case did the fracture involve the L3 vertebral body, and in this case a texture analysis of the adjacent non-fractured L4 vertebral body was exceptionally performed.

Twenty-two GLCM texture features were extracted from the L3 vertebral bodies using the radiomic IBEX platform (21) (version 1.0, http://bit.ly/IBEX_MDAnderson). The images were post-processed in 8 bit-depth for the calculation of GLCM features. Volumetric (3D) texture measurements were performed in 13 directions and 1 offset. The gray level intensities varied between 0 and 255, totalizing 256 gray levels. Mean and standard deviation of the measurements of each feature obtained in the 13 directions were calculated for statistical analysis. The following texture features were evaluated: autocorrelation, cluster prominence, cluster shade, cluster tendency, contrast, correlation, difference entropy, dissimilarity, energy, entropy, homogeneity1, homogeneity2, information measure corr1, information measure corr2, inverse diff moment norm, inverse diff norm, inverse variance, max probability, sum average, sum entropy, sum variance, and variance.

### Statistical analysis

SAS software (Statistical Analysis System, version 3.1.0, USA) and GraphPad Prism program (version 5.0. USA) were used for statistical analysis. Means±SD were calculated for continuous quantitative variables and the difference between the groups were analyzed using ANOVA and Duncan's post-test. The correlation between BMD and bone texture features was evaluated using Spearman's correlation coefficient (ρ) with determination of R value. P-value below 0.05 was set for statistical significance with a 95% confidence interval (CI).

Sample size calculation was performed based on results of a pilot study. A minimum sample size of 12 volunteers per study group was determined for a sample power of 80%.

## Results

### Clinical and radiographic features

There was no difference between groups regarding age, body mass index (BMI), and age of menopause as demonstrated in Table 1.

**Table 1:** Clinical characteristics of the study groups.

Groups	Age (years)	Body mass index (kg/m^2^)	Age at menopause (years)
Normal bone mass (control) (n=16)	69.94±4.48	27.47±3.01	49.56±4.66
Osteopenia without VFF (n=12)	67.92±5.53	27.70±4.09	48.67±5.71
Osteopenia with VFF (n=12)	74.17±5.57	27.82±5.22	46.00±3.71
Osteoporosis without VFF (n=12)	70.33±6.51	26.02±3.67	49.58±5.63
Osteoporosis with VFF (n=12)	72.58±5.30	27.32±4.12	46.42±4.05

Data are reported as means and standard deviations. VFF: vertebral fragility fracture; n: number of participants in each group.

The majority of VFF in the osteopenia and osteoporosis groups were classified as grade 1 and 2 and occurred in the lower thoracic spine or in the upper lumbar spine. Usually, the volunteers had more than one vertebral body affected by these types of fractures. None of the volunteers from the control group were affected by VFF. The distribution and grade of the VFF are demonstrated in Table 2.

**Table 2:** Position and grade of vertebral fragility fractures in the osteopenia and osteoporosis groups.

Volunteer	Osteopenia			Osteoporosis	
	Thoracic spine (T)	Lumbar spine (L)		Thoracic spine (T)	Lumbar spine (L)
V1	T4(1)	0		T6(1)	0
V2	T5(2), T6(2), T12(1)	0		T11(1)	0
V3	0	L1(1)		T4-T9(1)	0
V4	T10(1), T11(1), T12(1)	L1(1), L2(2), L3(2)		0	L4(1)
V5	T12(2)	L1(3), L5(1)		0	L1(3)
V6	T12(1)	0		0	L5(1)
V7	0	L5(1)		T11(1), T12(1)	L1(3)
V8	0	0		T10(2)	0
V9	0	L1(3), L5(1)		0	L4(1), L5(1)
V10	T11(1), T12(2)	0		0	L1(2)
V11	0	L1(1)		T12(2)	L1(2)
V12	0	L2(2)		T10(1), T11(2)	0

V: volunteer with fracture. The numbers in parentheses indicate the level of the vertebral fracture assessed by the semiquantitative visual technique of Genant, being 1 (mild), 2 (moderate), and 3 (severe).

### BMD and T-score of the lumbar spine (L1-L4)

The comparison between women with and without fractures did not show a significant difference in BMD and T-score (P>0.05). Means and standard deviations of BMD and T-scores of the groups are shown in Table 3.

**Table 3:** Bone mineral density (BMD) and T-score of the volunteers.

	BMD (g/cm^2^)	T-score
Normal bone mass (control)	1.05±0.08	-0.08±0.75
Osteopenia without VFF	0.85±0.05*	-1.76±0.42^#^
Osteopenia with VFF	0.86±0.07*	-1.85±0.35^#^
Osteoporosis without VFF	0.72±0.05*	-3.14±0.60^#^
Osteoporosis with VFF	0.69±0.06*	-3.37±0.49^#^

Data are reported as means and standard deviations. There were no significant differences in BMD or T-score between groups with and without VFF. Osteopenia and osteoporosis groups had lower BMDs and lower T-scores than the control group (*^#^P<0.05 compared to control, ANOVA). VFF: vertebral fragility fracture.

### MRI texture features

**Figure 2:**
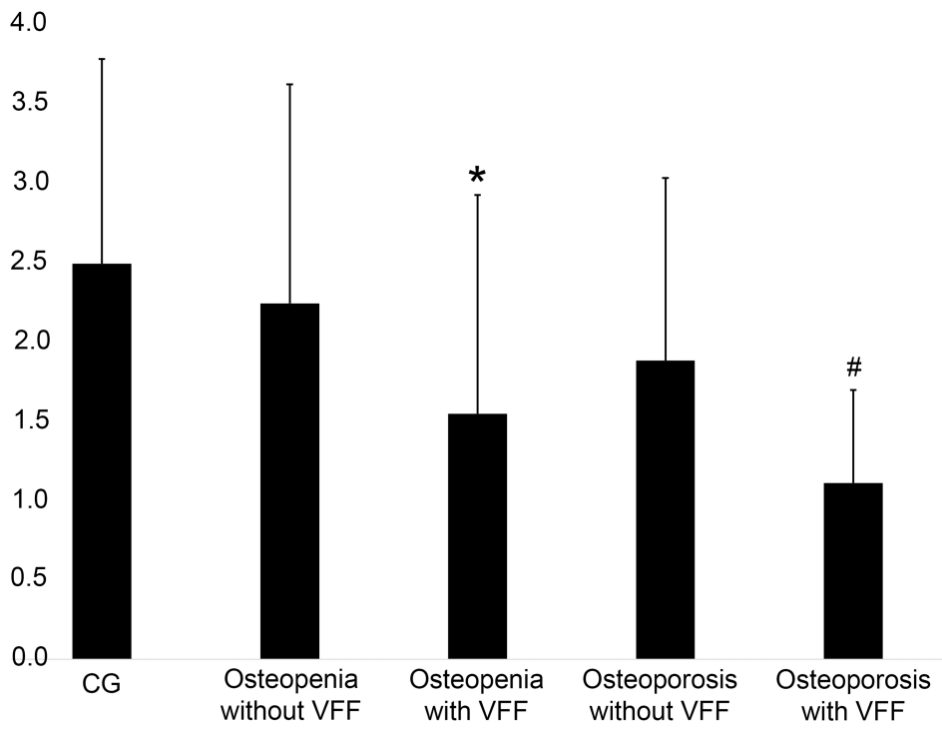
The gray level co-occurrence matrix cluster tendency feature extracted from the L3 vertebra in absolute numbers. *^#^P<0.05, with VFF compared to without VFF (ANOVA). CG: control group; VFF: vertebral fragility fractures.

**Figure 3:**
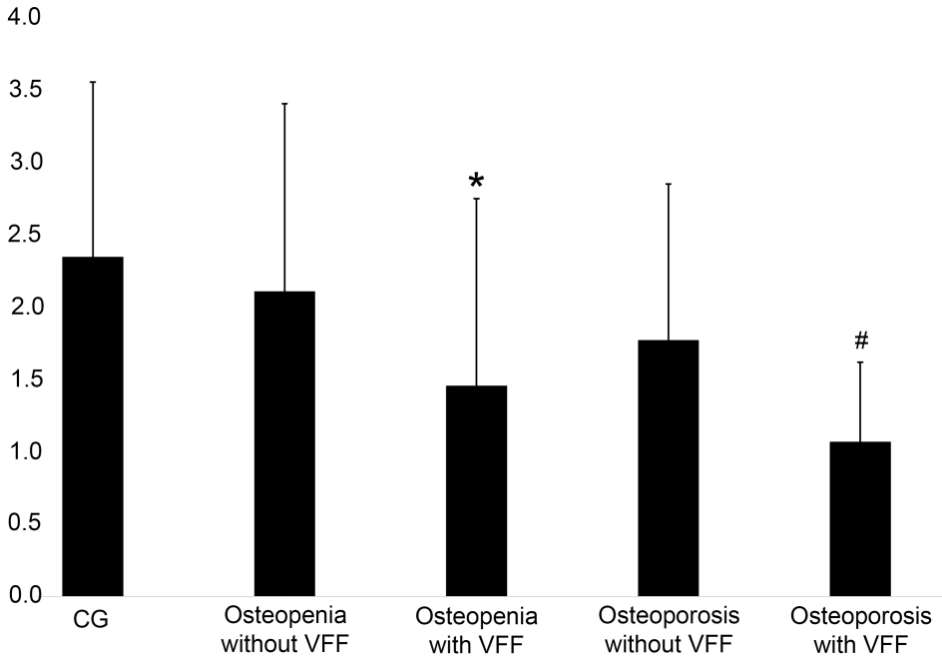
The gray level co-occurrence matrix cluster variance feature extracted from the L3 vertebra expressed in absolute numbers. *^#^P<0.05, with VFF compared to without VFF (ANOVA). CG: control group; VFF: vertebral fragility fractures.

Cluster tendency was significantly lower in the groups with VFF (osteopenia with VFF: 1.54±1.37, osteoporosis with VFF: 1.11±58) compared with the groups without VFF (osteopenia without VFF: 2.23±1.38, osteoporosis without VFF: 1.88±1.14; P=0.0103). Similarly, variance was significantly lower in the groups with VFF (osteopenia with VFF: 1.44±1.37, osteoporosis with VFF: 1.13±59) compared with the groups without VFF (osteopenia without VFF: 2.34±1.38, osteoporosis without VFF: 1.89±1.14; P=0.0113). These results are presented in Figures 2 and 3.

We observed a positive correlation between BMD and cluster prominence (r=0.409), cluster tendency (r=0.345), correlation (r=0.570), entropy (r=0.364), information measure corr1 (r=0.378), inverse variance (r=0.449), sum entropy (r=0.320), and variance (r=0.338) and a negative correlation between BMD and sum average (r=-0.274) and sum variance (r=-0.266) [P<0.05]. Table 4 shows the results of Spearman's correlation analysis between the BMD and the significant bone texture features.

**Table 4:** Spearman's correlation coefficient between bone mineral density (BMD) and bone texture features.

Bone texture feature	Rho for correlation with BMD	P-value
Cluster tendency	0.345	0.001
Cluster prominence	0.409	0.006
Correlation	0.570	<0.001
Entropy	0.364	0.003
Information measure corr1	0.378	0.002
Inverse variance	0.449	<0.001
Sum entropy	0.320	0.011
Variance	0.338	0.007
Sum average	-0.274	0.030
Sum variance	-0.266	0.035

## Discussion

Texture features extracted from routine T2-weighted MRI were able to discriminate between women with and without VFF. The features cluster tendency and variance had significantly lower values in fractured women compared to non-fractured women. In addition, the features cluster prominence, cluster tendency, correlation, entropy, information measure corr1, inverse variance, sum entropy, variance, sum average, and sum variance presented a significant correlation with BMD. Our results demonstrated the potential use of routine MRI sequences to assess bone fragility and osteoporosis.

Texture features characterize the spatial distribution of gray intensities within a region or volume of interest. The GLCM method determines from an image/volume the probability of occurrence of pixel/voxel pairs of intensities, given a distance d and an orientation θ (16). Texture analysis using this method allows the identification of patterns in the images that are not identified by the human eye and basically reflect the heterogeneity of the tissue, which can be used as a biomarker for diagnosis and prognosis purposes in several pathological processes (16).

A study published by Burian et al. (22) demonstrated the heterogeneity of bone marrow with bone mass loss. In this study, the authors extracted texture features from chemical-shift MRI sequences of pre-menopausal women and compared them with those of post-menopausal women, demonstrating that the features dissimilarity and contrast were able to differentiate between the groups. Bone mass loss in osteoporosis determines signal heterogeneity in bone marrow on MRI protocols. Although this signal heterogeneity can be detected by visual evaluation, it does not allow the diagnosis of osteoporosis or the grading of its severity. In this context, opportunistic radiomic texture analysis from MRI sequences could be used as a new noninvasive tool to assess skeletal fragility.

A previous study evaluated the inter- and intra-observer reproducibility of bone texture features extracted from routine lumbar spine MRI using the IBEX radiomic platform (17). In that study, the authors demonstrated an excellent inter- and intra-observer reproducibility for the GLCM texture features using two different segmentation methods. In addition, the authors demonstrated a significant correlation between BMD and thirteen texture features and found no significant difference when comparing the two segmentation models. However, this previous study did not address the correlation between bone texture and fractures. A study published by Zaworski et al. (23) correlated fragility fractures with bone texture features extracted opportunistically from lumbar spine MRI. In this study, the authors demonstrated that patients with fragility fractures had more texture variability and disorder and less homogeneity compared with controls.

Our texture analysis from routine lumbar spine MRI showed that the features variance and cluster tendency were able to differentiate between women with and without fractures. The cluster tendency feature evaluates the proportion of voxel groupings with similar gray-level values while the variance feature evaluates the variance in grey level across pixels. Both features reflect the level of homogeneity within a region of interest and quantify the gray level variability between neighboring pixels (24-27).

The results of the mentioned studies and our results reinforce the potential use of routine MRI sequences to extract parameters that may be correlated with skeletal fragility and bone loss, enabling early detection of osteoporosis.

The current investigation had limitations. First, we limited our analysis to L3 vertebra. Only one volunteer (number 4) had texture analysis performed on L4 vertebra, due to a fracture in L3. We chose to evaluate the L3 vertebral body because L1 and L2 are usually more affected by fragility fractures, while L4 and L5 vertebrae are most affected by degenerative changes in their endplates, and such abnormalities may affect texture analysis and work as confounding factors. Also, we extracted the texture parameters through the positioning of a circular ROI in the center of the vertebra trying to avoid the degenerative changes on their endplates. The extraction of features by segmentation of the entire vertebral body excluding the cortical bone needs to be tested in future studies.

Another limitation of our study was that only FSE T2 weighted sequences were used for texture feature extraction. Qualitative evaluation of T1 weighted images is considered essential for bone evaluation in the clinical practice, and we did not test the potential of this sequence for quantitative analysis. We used the T2 weighted sequence because it had already been tested in a previous study, in which the extraction of features proved to be reliable and some extracted features correlated with BMD (17). We suggest that T1 weighted sequences should be evaluated in future research to assess whether the potential of using MRI radiomic attributes or biomarkers can be increased.

In conclusion, the current study highlights the potential use of routine MRI sequences to identify biomarkers of skeletal fragility. While independent studies with larger sample sizes are still needed to validate this method, our findings reinforce the importance of multiparametric assessment of bone quality for fracture risk evaluation and encourage future studies using MRI.
